# Health Services Use and Costs in Individuals with Autism Spectrum Disorder in Germany: Results from a Survey in ASD Outpatient Clinics

**DOI:** 10.1192/j.eurpsy.2022.1072

**Published:** 2022-09-01

**Authors:** J. Höfer, F. Hoffmann, M. Dörks, I. Kamp-Becker, C. Küpper, L. Poustka, S. Roepke, V. Roessner, S. Stroth, N. Wolff, C. Bachmann

**Affiliations:** 1 Carl von Ossietzky Universität, Dept. Of Health Services Research, Oldenburg, Germany; 2 Philipps-Universität Marburg, Dept. Of Child & Adolescent Psychiatry, Marburg, Germany; 3 Charité - Universitätsmedizin Berlin, Dept. Of Psychiatry, Berlin, Germany; 4 Universitätsmedizin Göttingen, Dept. Of Child & Adolescent Psychiatry, Göttingen, Germany; 5 Medical Faculty of the Technical University Dresden, Dept. Of Child & Adolescent Psychiatry, Dresden, Germany; 6 Universitätsklinikum Ulm, Dept. Of Child & Adolescent Psychiatry, Ulm, Germany

**Keywords:** health services, autism, Germany, costs

## Abstract

**Introduction:**

Autism spectrum disorders (ASD) are associated with high services use, but European data on costs are scarce.

**Objectives:**

Utilisation and annual costs of 385 individuals with ASD (aged 4-67 years; 18.2% females; 37.4% IQ < 85) from German outpatient clinics were assessed.

**Methods:**

Client Service Receipt Inventory

**Results:**

Average annual costs per person were 3287 EUR, with psychiatric inpatient care (19.8%), pharmacotherapy (11.1%), and occupational therapy (11.1%) being the largest cost components. Females incurred higher costs than males (4864 EUR vs. 2936 EUR). In a regression model, female sex (Cost Ratio: 1.65), lower IQ (1.90), and Asperger syndrome (1.54) were associated with higher costs.

**Conclusions:**

In conclusion, ASD-related health costs are comparable to those of schizophrenia, thus underlining its public health relevance. Higher costs in females demand further research.

**Disclosure:**

No significant relationships.

